# Visual short-term memory, culture, and image structure

**DOI:** 10.3758/s13414-025-03094-7

**Published:** 2025-05-27

**Authors:** Huilin Li, Jessie Chien, Angela Gutchess, Robert Sekuler

**Affiliations:** Department of Psychology, Brandeis University, 415 South Street, MS 062, Waltham, MA 02454 USA

**Keywords:** Short-term memory, Cultural differences, Perceptual process, Spatial frequency, Image structure

## Abstract

Cultural differences in cognition, including visual perception and long-term memory, may arise because typical visual environments differ across cultures, particularly in their spatial scale. Consequently, the influence of culture on cognitive processing depends on whether stimuli are presented at a large or small spatial scale. We tested North American and East Asian young adults to determine whether such cultural differences extend to short-term memory—testing, for the first time, whether spatial frequency information contributes to cross-cultural differences in memory. Test materials were images of natural and constructed scenes whose spatial structure was manipulated by low-pass filtering. Several seconds after briefly viewing a target scene, a subject saw three versions of that scene: the target itself and two variants whose low-pass filtering differed from the target. From these three, the subject selected the image identical to the target. The two groups did not differ in overall recognition accuracy but did in the way they mistook nonmatching images for certain targets. Specifically, North American subjects made reliably fewer errors in matching images whose high-frequency content was intact, providing evidence that cultural differences in prioritization of high spatial frequency information extend to short-term memory. Across both groups, subjects were highly accurate at recognizing images that retained all or most of their high-spatial frequency content and were highly sensitive to different levels of spatial filtering. These findings show that visual memory has sufficient fidelity to support fine discrimination of variation in spatial frequency.

**Supplementary Information:**

The online version contains supplementary material available at 10.3758/s13414-025-03094-7.

## Introduction

Differences in genes (Chiao, 2011), norms, practices, and physical environments contribute to cultural differences in various forms of cognition (Gutchess & Sekuler, 2019). Among other effects, these differences impact visual processing and, therefore affect memory for what was seen. Recent behavioral studies revealed cultural differences in responses to stimuli thought to be processed at different levels of the visual hierarchy (e.g., Blais et al., 2021). Everyday visual scenes typically contain information spanning a wide range of spatial frequencies, and the processing of this information can be selectively modulated by expectation, attention, and other top-down factors (Flevaris et al., 2011; Groen et al., 2017; Shulman & Wilson, 1987; Sowden & Schyns, 2006). The potential for top-down control over the use of different spatial frequency channels could produce differences across cultures in which frequency bands tend to be prioritized. For example, psychophysical studies with images of faces showed that East Asians tend to prioritize low spatial frequency information, which is associated with coarser, more global information, whereas Westerners tend to prioritize higher spatial frequency information associated with fine details and local features (Blais et al., 2021; Caldara et al., 2010; Estéphan et al., 2018; Im et al., 2017; Kelly et al., 2010; Miellet et al., 2013; Rodger et al., 2010; Tardif et al., 2017). Tests with lower-level, simple stimuli, such as sinusoidal gratings or Gabor patches, produce a different outcome. In particular, the contrast sensitivity functions of Caucasian and East Asian subjects seem not to differ (Tardif et al., 2017), and N1 and P3 cortical potentials evoked by Gabor patches of higher or lower spatial frequencies are similar for Americans and East Asians (Lin et al., 2022). This latter null result is important because both these cortical potentials are known to exhibit strong spatial frequency dependence.

Accurate memory for visual stimuli requires that image content be processed and maintained in memory with minimal loss. Particularly when the memory assay involves recognition or match to sample, performance depends upon the quality of additional processing steps, including multiple retrieval processes. Recent research has begun to probe the quality of visual memories. Rivera-Aparicio and colleagues (2021) tested short-term memory for full-color images with various blur levels. Participants were recruited through Amazon Mechanical Turk (mTurk); their cultural background was not specified, but the majority of mTurk workers are located in the USA. The experimenters found that people tended to remember images as more vivid—that is, as having less blur, as well as more colorful (i.e., more saturated) and as higher resolution (i.e., less pixelated). Using a different paradigm to test long-term memory, Cooper and colleagues (2019) found that people remembered images as being less visually salient. Although the precise qualities and vividness of visual memories will undoubtedly be impacted by the content and visual features of stimuli, as well as the length of delay, these findings are relevant given our interest in studying the influence of culture on memory for spatial frequency information.

To produce sensitive tests of visual memory accuracy, we generated stimuli whose spatial content had been manipulated by different levels of low-pass filtering, which diminishes high-frequency details. We used these challenging materials to examine possible cultural effects in visual memory, an ability that occupies the intersection of perceptual and mnemonic processes (e.g., Bainbridge, 2019). Previous research showed cultural differences in long-term visual memory (for reviews, see Gutchess & Sekuler, 2019; Wang, 2021). Because errors in visual memory can occur even after very short time delays, on the order of seconds or less (Atkins & Reuter-Lorenz, 2008; Chunharas et al., 2022; Huang & Sekuler, 2010), we tested whether cultural differences extend to visual memory at very short timescales (a few seconds).

For test materials, we chose images of natural and constructed scenes. Perception of scenes recruits visual processes beyond low-level vision, such as those engaged when viewing isolated objects such as faces or simple stimuli such as gratings. Relative to other visual stimuli, a scene’s distinguishing characteristics can be described as “a semantically coherent (and often nameable) view of a real-world environment comprising background elements and multiple discrete objects arranged in a spatially licensed manner” (Henderson & Hollingworth, 1999). A scene’s visual content spans a range of spatial frequencies (Blake & Sekuler, 2006), with background elements usually, but not always, larger scale and its objects smaller scale (Võ, 2021). Consistent with the idea that higher spatial frequencies typically signal a scene’s object-like elements, patterns with relatively higher-frequency content tend to be perceived as figures rather than ground (Klymenko & Weisstein, 1986). Beyond these basic perceptual effects, culture can also shape how observers allocate attention to local versus global information, as Masuda and Nisbett (2006) demonstrated in a multi-experiment study. In that study, East Asians and Americans were presented with scenes in which either local, object information or large-scale, contextual information had been changed. The dependent measures were the subjects’ speed and accuracy in detecting change between the two versions of the image. Compared with American subjects, East Asians more readily detected contextual, large-scale, rather than focal, changes, suggesting culture-based variation in how visual attention prioritizes information in a scene.

To examine possible cross-cultural differences in short-term memory, we manipulated the spatial content of scenes that subjects saw. We defined cultural groups based on national origin, drawing on the East/West differences that have been a focus of the literature (e.g., Nisbett & Masuda, 2003). We preregistered predictions that cultural groups would differ in their dependence upon and attention to different spatial frequency bands, with Americans relying on high-spatial frequency (HSF) information relatively more than Asians, and vice versa for low-spatial frequency (LSF) information. Applying a low-pass spatial filter to real-world scenes reduces the available high-frequency content with little effect on the low-frequency content. This led us to predict that cultural differences in visual memory should emerge for the scenes that contain the most high-frequency information. As the amount of high-frequency content is reduced through filtering, the memory performance of Americans should be impacted disproportionately compared with East Asians. These predictions align with research showing that East Asians tend to prioritize lower-frequency information, whereas Westerners tend to prioritize higher-frequency information (reviewed by Blais et al., 2021). Because scenes’ object-related information tends to lie in higher-frequency bands, that finding is consistent with reports that Westerners attend to objects relatively more than East Asians (Chua et al., 2005; Masuda & Nisbett, 2001). Following that, we predicted that compared with East Asians, Americans would have more accurate memories of images containing the highest spatial-frequency information.

We compared cultural groups using a short-term visual memory task to test these predictions. Americans and East Asians viewed grayscale scenes that were low-pass filtered to different degrees. The filtering operation produced images that differ mainly in high-spatial-frequency information. In the first stage of a delayed-match-to-sample (DMS) design, subjects first saw an image of a scene; then, after a random masking stimulus and a short delay, they saw three variants of that scene, which differed in high-frequency content. Subjects tried to identify the image that matched the one they had seen initially. The modest spatial frequency differences and high interitem similarity among stimuli added to the challenge of the subjects’ task. The perceptually similar alternatives on each DMS trial made the task akin to a multiclass classification (Sokolova & Lapalme, 2009). This allowed us to go beyond the measure commonly used in DMS research, the proportion of correct matches, to examine the precision of subjects’ classification at various levels of spatial filtering. These challenging test stimulus alternatives afforded us more information than would normally be available from a DMS design.

Our experimental design also included test images’ memorability as a variable. Research has begun to identify the features that make images memorable, including semantics, scene category, the presence of people, and particular objects, such as cars or seats (Bainbridge, 2019; Isola et al., 2014; Rust & Mehrpour, 2020). Isola et al.’s (2014) study defined memorability by how often subjects correctly detected the repetition of a briefly presented image. Repetitions were separated by varying numbers of non-repeating, filler images. Using a subset of Isola et al.’s images, we assessed whether scenes that vary in memorability are differentially impacted by manipulations of their spatial frequency content. Beyond testing the effects of culture, this manipulation allowed us to assess how both spatial frequency and memorability manipulations affect memory performance across the entire sample.

## Method

### Subjects

We recruited 60 subjects, 30 US-born and 30 East Asian-born Brandeis University students. All the American subjects had spent most of their lives in the USA: two had spent 4 or fewer years living abroad, and the rest had lived their entire lives in the USA. The East Asian subjects had all spent less than 5 years in the USA, on average just 2.19 years (*SD* = 1.32) in the USA and 17.88 years (*SD* = 1.38) in their native country (China for 29/30, N/A for the remaining participant). Of the Americans, ten were men, 19 were women, and one was nonbinary/other; East Asians included one man and 29 women. Subjects’ age range was 18–23 years (*M* = 20.2, *SD* = 2.2). Age data are missing for two East Asian subjects, and vision measures (contrast sensitivity and logMAR, a measure of visual acuity) are missing for one. Visual acuity was measured using the Sloan ETDRS Intermediate Vision chart (at 60 cm). Contrast sensitivity was measured using the Mars Perceptrix Letter Chart for near vision. Finally, because of the high incidence of myopia in East Asia, including China (Dolgin, 2015), we asked about the subjects’ refractive condition and history. Self-reports indicated that 15/30 Americans and 22/29 East Asians (data were missing for one participant) were nearsighted; myopic Americans reported that they began to wear corrective lenses at *M* = 11.40 (*SD* = 3.83) years of age; myopic East Asians began at a similar age, *M* = 11.94 (*SD* = 2.93) years of age. Table 1 gives additional details about the demographics of our subjects.

**Table 1:** Selected comparisons between groups (M and SD)

	Americans	East Asians	*t* value	*p* value
Age	19.63 (1.33)	19.96 (1.14)	1.02	0.31
Contrast sensitivity	1.79 (0.07)	1.81 (0.05)	1.01	0.32
logMAR Acuity	− 0.20 (0.05)	− 0.19 (0.06)	0.80	0.43

After the experiment, subjects were given an online questionnaire of 15 demographic questions about age, gender, education level, ethnicity, race, native country, native language, years in the USA, and optical refraction, if any. They also completed the Analysis-Holism Scale (AHS; Martin-Fernandez et al., 2022), which examines systematic cognitive differences regarding holistic–analytic thinking style, and 30 questions from the Self-Construal Scale (SCS; Singelis, 1994), which measures tendencies toward independence and interdependence. Results are summarized in Table 2. Note that scores are missing for one East Asian subject who failed to complete the scales. Questionnaires were administered to characterize our samples and to either establish comparability (e.g., on age, measures of vision) or assess cultural differences (e.g., measures of values). We will return to the significance of these results in the Discussion.

**Table 2:** Self-Construal Scale (SCS) and Analysis-Holism Scale (AHS) results (*M* and *SD*)

Measure	Americans	East Asians	*t* value	*p* value
SCS-independence	4.72 (0.69)	4.65 (0.65)	0.39	0.70
SCS-interdependence	4.88 (0.65)	4.67 (0.69)	1.21	0.23
AHS-total	120.57 (9.34)	117.62 (10.94)	1.11	0.27

### Stimuli

**Fig. 1:**
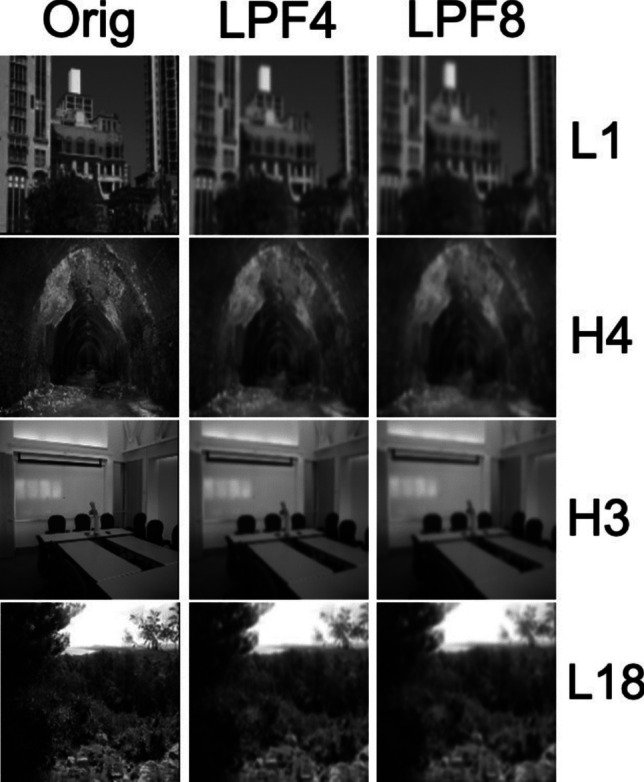
The rows show four representative stimuli categorized as high memorability (H) and low (L). In each row, the original image and its two low-pass variants are shown from left to right columns. We computed the mean Structural Similarity Index (SSIM; Z. Wang et al., 2004), which compares images based on luminance, contrast, and structural information—all key components of human visual perception. When two images are identical (perfect copies of one another), SSIM = 1; when SSIM = 0 the images’ structure, luminance, and contrast differ to the extent that the algorithm can find no commonality. Across all images, the mean SSIM for Orig vs. LPF4 and LPF4 vs. LPF8 images are 0.5968 and 0.7514, showing that images in the leftmost two columns are more different than those in the rightmost columns

Stimuli were 40 images of natural and indoor scenes selected from the Scene Understanding (SUN) dataset of 131,000 images distributed across multiple categories (Xiao et al., 2010). Our selection of images excluded ones in which a human face, body, or other identifiable living creature appeared. Using Isola et al.’s (2011) image memorability metric, we selected equal numbers of high- and low-memorability images. This allowed us to assess how image memorability affected subjects’ performance on our task. High memorability was defined by memorability scores > 0.7, while low memorability was defined by scores < 0.3. The lefthand column of Fig. 1 shows representative images of both types.

MATLAB’s Image Processing Toolbox converted the images to 8-bit grayscale. Two variants were then produced for each original grayscale image by convolving the image with a low-pass 3 × 3 normalized box filter. Each convolution reduced the high-frequency content of the image, leaving its low-frequency content essentially unchanged. The first variant was produced by four iterative applications of the convolution kernel; to remove additional high-frequency content, a second image variant was iteratively filtered four more times, making eight iterative convolutions. We designate members of each trio of images as Orig, LPF4, and LPF8 for the original unfiltered image and images that were iteratively low-pass filtered four and eight times, respectively. For reference, these filtering operations correspond closely to what would be produced by a single application of Gaussian filters, with σ = 2 pixels or σ = 2.83 pixels for LPF4 and LPF8, respectively (Gonzalez & Woods, 2017). The values for the average blur radius in pixels are ~ 6 and ~ 9. The difference in stimulus blur between Orig and LPF4 will be larger than between LPF4 and LPF8, which should produce a corresponding perceptual difference in stimulus discriminability (Watson & Ahumada, 2011), making DMS more challenging for comparisons with smaller perceptual differences.

Figure 1 shows examples of four images produced by these filtering operations. The low-pass filtering protocol, including the design of the low-pass filter and its iterative application, was based on pilot testing with four subjects, none of whom would serve later in our experiment. Pilot testing identified levels of filtering that would produce accuracy well above chance but below the upper limit of accuracy.

**Fig. 2:**
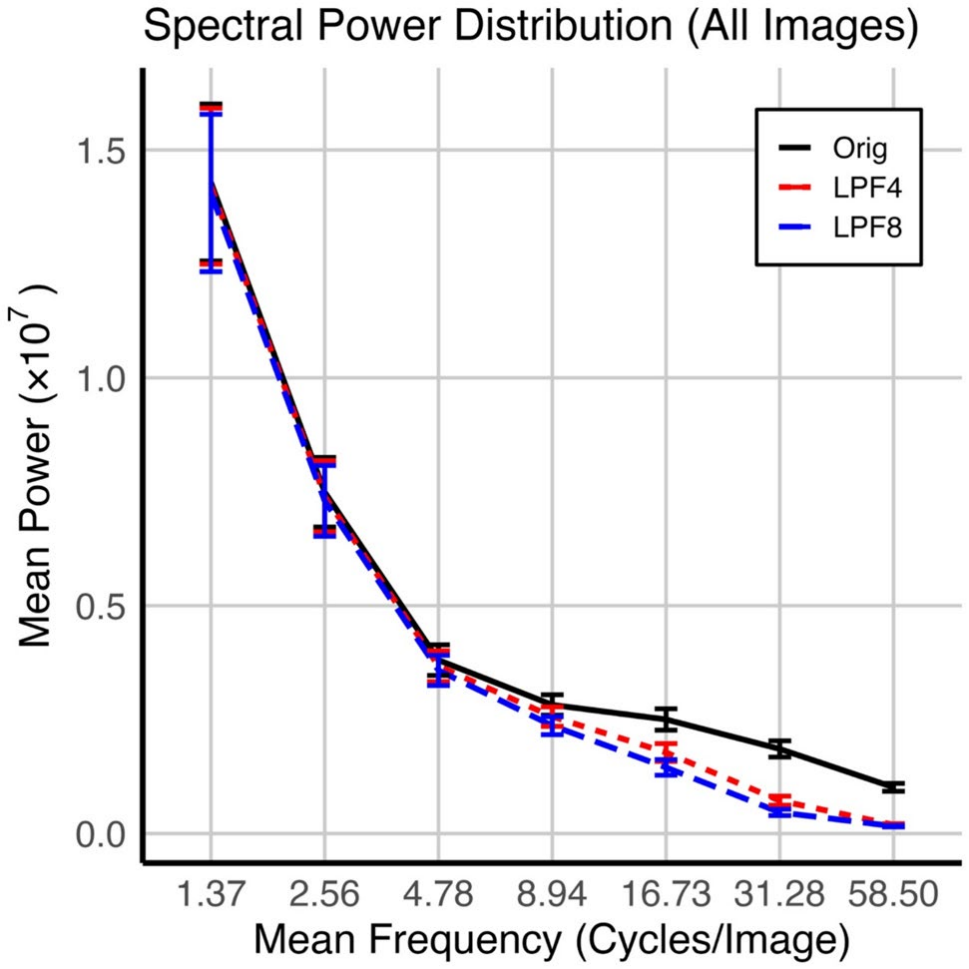
Radial frequency spectra of the three types of image variants (Orig, LPF4, LPF8). Each point represents the summed spectral power within a given frequency bin, averaged across images of the same low-pass filter level. The *x*-axis represents the mean spatial frequency per bin (cycles/degree), while the *y*-axis shows summed power in arbitrary units. Error bars indicate the mean (*SEM*) standard error across images

Repeated application of the filter reduces an image’s high-frequency content with diminishing effect over successive iterations, while essentially having no effect on the lower frequencies in the image. This is demonstrated in Fig. 2, which shows the mean spatial frequency content for each variant of a test image, the Orig, LPF4, and LPF8 variants. We computed its radial frequency spectrum and then averaged across images at each level of filtering. Specifically, each image variant was subjected to a 2D Fast Fourier Transform (FFT) to obtain its frequency representation. The power spectrum was then computed as the squared magnitude of the FFT coefficients. The resulting frequencies were aggregated into seven logarithmically spaced radial bins. Power values within each bin were summed and averaged to generate the radial frequency distribution for each image.

We used R’s *emmeans* package to quantify how the filtering operations affected images’ frequency content. Working with the data summarized in Fig. 2, we carried out paired comparisons among the image variants for each frequency bin shown in the figure. After Bonferroni correction for 21 comparisons, no paired comparisons for the lowest three mean frequency bins were statistically significant (all *p* values > 0.05), while all other paired comparisons save one were significant (all *p* values < 0.05). The only exception was the comparison between LPF4 and LPF8 at the highest frequency bin (*p* > 0.50). We believe that comparison failed because both items were near zero.

### Procedure

Subjects gave written informed consent for a protocol approved by Brandeis University’s Institutional Review Board. Their visual acuity and contrast sensitivity were then tested using the EDTRS visual acuity test (VectorVision, Houston, TX) designed for a 60-cm test distance and the Mars Letter Contrast Sensitivity Test (The Mars Perceptrix Corporation, Chappaqua, NY). Stimuli were presented on a Dell Flat Panel LCD Monitor (P2011HT) with a screen resolution of 1,600 × 900 pixels (over 18.22 × 10.71-in.) and a refresh rate of 60 Hz. The monitor had a constant luminance of 35.4 cd/m^2^. The scenes represented in our images varied in subject matter and mean luminance, which ranged from 21 to 55 cd/m^2^; at the viewing distance of 32 inches, each image subtended 5.1° visual angle.

**Fig. 3:**
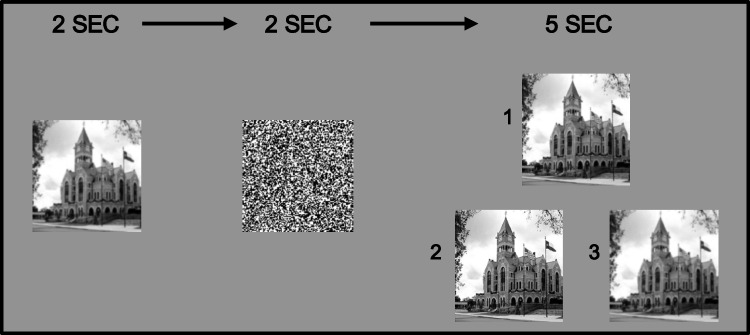
Example trial flow shown from left to right. Subjects first saw the trial’s target image for 2 s, followed by a 2-s-long masking stimulus. Next, they were presented with three test images that varied in level of filtering; one was the target image. The subjects’ task was to select the test image (labeled 1, 2, or 3) that matched the target image shown at the start of the trial, taking no more than five seconds to click on the image

The experimental task, which was implemented in PsychoPy (Peirce et al., 2019), entailed a DMS design. Figure 3 shows our task’s sequence of events on a trial. First, a sample target stimulus (e.g., an image of a scene) was displayed for 2-s. A 2-s presentation of a random mask followed this. Finally, with three variants of the target image on the display screen, a subject had to identify the one that matched the remembered target image. Subjects indicated their selection by pressing the computer keyboard’s “1” key to indicate the top image, and the “2” and “3” keys to indicate the lower left or lower right image. They had up to 5 s to respond, and the program advanced to the next trial. After each set of 10 trials, subjects took a short self-paced break. While familiarizing themselves with the task, subjects were told they would “see a scene image and be tested on [their] memory for that exact image.” They were shown a sample target image, the masking stimulus, and examples of the three test images from which they would choose the exact match to the target image. After the instructions, subjects had two practice trials, receiving feedback after each response. During the actual experiment, no feedback was given after a trial.

Each subject completed 40 trials, distributed over four blocks of 10 trials each. This included approximately equal numbers of trials with the target image at each level of spatial filtering. The 40 trials entailed approximately equal numbers of target images at each of the three levels of filtering (13, 14, and 13 for 0 [the original image], 4, and 8 filtering operations, respectively); the comparison image that was the correct match appeared approximately often in each of the three possible positions on the screen (13 at top and bottom left, 14 at bottom right). Blocks were presented in a randomized trial order, but within a block, all subjects received target and comparison images in the same order. The primary dependent variable is accuracy—the proportion correct based on selecting the correct match from the three test images (see Fig. 3).

## Results

A programming error caused one image to be presented as the standard twice, at two different levels of filtering. All analyses excluded that trial, leaving 39 trials for each subject. In addition, during low-pass filtering, one exemplar from seven of the stimuli acquired a small edge artifact, a thin white line at one of the image’s edges.

We first wanted to see if memory performance with the seven images with edge-artifact differed from performance with the other images. Mean accuracy and 95% confidence intervals (CIs) were 0.738 [0.655, 0.821] and 0.730 [0.693, 0.766] for the seven images with artifacts and the remaining images, respectively. The very small (0.008) difference between the two means suggests that edge artifacts did not significantly affect accuracy, so subsequent analyses included both sets of images.

Categorization of images as high or low in memorability was based on psychophysical estimates with full-color versions of our grayscale images, and assessed with a protocol quite different from ours (Isola et al., 2014). These differences led us to entertain the possibility that the variable might be unimportant for our results. To test that, we calculated the mean proportion of each subject’s correct responses for high and low memorability test items to assess the importance of image memorability categories. The mean and 95% CIs are shown in Table 3. A paired *t* test confirmed that the difference in memorability did not reliably impact performance (*t* = 0.12, *df* = 59, *p* = 0.9). As a result, subsequent analyses omitted image memorability as an independent variable.

**Table 3:** Memory accuracy for target stimuli drawn from high- and low-memorability images (based on Isola et al., 2014)

Category	Mean	Lower CI	Upper CI
High memorability	0.671	0.644	0.698
Low memorability	0.668	0.641	0.696

**Fig. 4:**
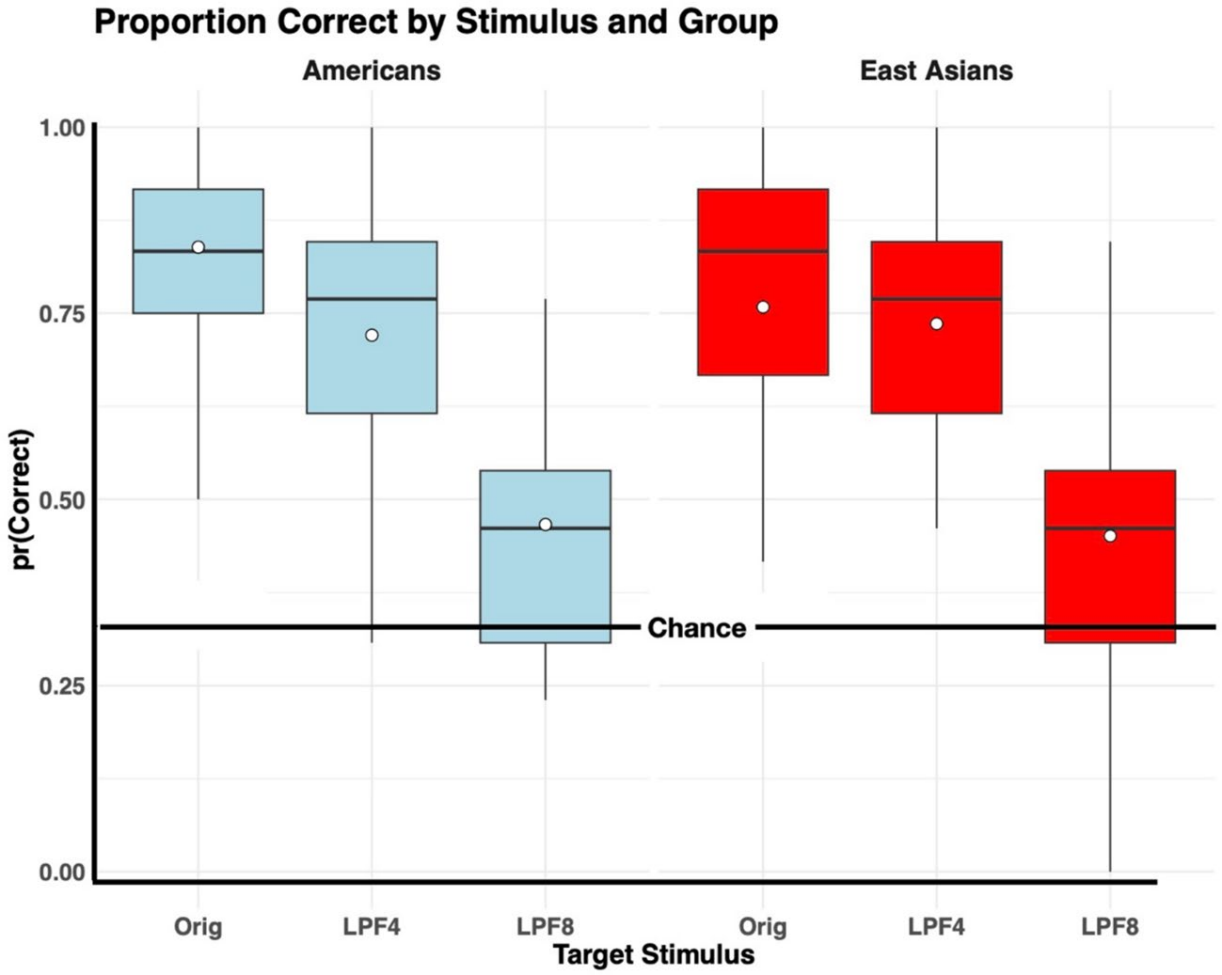
The proportion correct for each combination of group (Americans, East Asians) and targetStimulus (Orig, LPF4, LPF8). The white dot inside each box indicates the data’s mean

Next, we turned to our main interest, cultural differences, and their possible interaction with the to-be-remembered target image’s spatial frequency content. We found the proportion of correct responses of East Asian and American subjects for each of the three target image filter levels. In Fig. 4, these are summarized as box-and-whisker diagrams. Inside each box, the median, represented by a horizontal line, is supplemented by a white dot representing the mean.

The values plotted in the figure were then entered into a linear mixed-effects analysis of variance (ANOVA) using R’s *lme4* package (Bates et al., 2015). Group (that is, culture) was a between-subjects variable, and the target stimulus was a within-subject factor. The results are shown in Table 4, where “targetStimulus” refers to whether the target stimulus had been filtered zero, four, or eight times. Results show that filter level was a significant source of variance in subjects’ performance, but neither culture nor culture’s interaction with filter level was.

**Table 4:** Summary of ANOVA on mean proportion correct responses

Effect	*df*	*F*	*p* value
targetStimulus	2, 116	67.41	< 0.001
Group	1, 58	1.55	0.220
targetStimulus:Group	2, 116	1.12	0.330

For a closer look at how performance differed with the targetStimulus’ level of filtering, we used R’s *emmeans* package to make paired comparisons between levels of target stimulus filtering, with data averaged over cultures. Table 5 shows the results, including the effect size (Cohen’s *d*) associated with each contrast. The differences among the contrast sizes are informative. Using the customary interpretation of *d* values, the contrast between Orig and LPF4 produced a small-to-medium effect, while the other two contrasts produced large effects. This confirms what we expected from differences among our images’ blur discriminability: The difference between Orig and LPF4 targetStimuli was appreciably smaller than between the other two contrasts.

**Table 5:** Pairwise comparisons between levels of targetStimulus

Contrast	Estimate	*SE*	*t* ratio	*p* value	Cohen’s *d*
Orig vs. LPF4	0.070	0.03	2.366	0.051	0.443
Orig vs. LPF8	0.326	0.03	11.028	< 0.0001	2.058
LPF4 vs. LPF8	0.256	0.03	8.661	< 0.0001	1.619

Results are averaged over the levels of group. *df* = 116 (Kenward–Roger method). *p* value adjustment used the Tukey method for comparing a family of three estimates. A positive estimate means that the contrast’s first term was larger than its second; a negative estimate means the opposite

Up to this point, we have concentrated on just one component of the DMS paradigm, the targetStimulus that subjects see first and must hold in memory. Of course, the elements that constitute a trial’s multiple test alternatives are equally important, particularly because of the relatively modest perceptual differences among the alternative images (see Fig. 1). Also, our analyses so far have concentrated on the binary categorization of DMS responses as “right” or “wrong.” Of course, the existence of wrong responses does not tell us everything about the underlying cause of errors; for additional information, we turned to how errors were distributed across stimulus variants. In doing this, we exploited perceptual similarity’s strong contribution to confusion in short-term memory, including confusions found in previous applications of the DMS paradigm (Schurgin et al., 2020; Sekuler & Sekuler, 2023; Yotsumoto et al., 2007). To examine how well subjects’ performance aligns with inter-item similarity, we sorted the proportion of responses, correct as well as incorrect, into the 3 × 3 stimulus–response confusion matrices shown in Table 6, one for each group.

**Table 6:** Confusion matrix for each group

Stimulus:	Orig	LPF4	LPF8
American subjects			
*Response:*			
* Orig*	**0.839**	0.074	0.023
* SeM*	*0.022*	*0.021*	*0.009*
* LPF4*	0.133	**0.721**	0.510
* SeM*	*0.021*	*0.028*	*0.033*
* LPF8*	0.028	0.205	**0.467**
* SeM*	*0.011*	*0.026*	*0.030*
East Asian subjects			
*Response:*			
* Orig*	**0.758**	0.049	0.031
* SeM*	*0.033*	*0.009*	*0.007*
* LPF4*	0.208	**0.736**	0.518
* SeM*	*0.031*	*0.030*	*0.033*
* LPF8*	0.033	0.215	**0.451**
* SeM*	*0.012*	*0.028*	*0.034*

For both groups, correct responses are represented along the matrix’s negative diagonal and are displayed in boldface; incorrect responses fill off-diagonal cells. Note that this way of representing subjects’ performance goes beyond the basic results expressed as proportion correct. They show how incorrect responses, confusions, are distributed among alternatives. We begin by examining the pattern of confusion common to both groups. Interestingly, this pattern of confusion parallels the images’ structural similarity (SSIM) values. Consider first Table 6’s lefthand column, which shows how responses are distributed when the target image is unfiltered. Subjects rarely confuse an unfiltered target image with either filtered test stimulus. That specific lack of confusion mimics the dissimilarity (lower SSIM value) of unfiltered images (Orig class) to images whose high spatial frequencies were reduced (either LPF4 or LPF8). Jumping to the righthand column, we see that the most strongly filtered target images (LPF8 class) are often confused with LPF4 test images, a result that aligns with the relatively high SSIM values between images of those two types. Finally, in the center column, we see that LPF4 target images are less likely to be confused with Orig alternatives than with the other (LPF8), which mimics the relative sizes of SSIM values for Orig and LPF4 images on the one hand, and those of LPF4 and LPF8 images on the other.

We now turn to the differences between results for the two groups. To uncover differences between the cultural groups in Table 6 and guided by our preregistered hypotheses, we made the paired comparisons shown in Table 7. Notably, cultural differences emerged for the stimuli containing the most high-frequency information. American subjects were more accurate at correctly recognizing high-frequency stimuli (Orig) than were East Asian subjects; in contrast, East Asians tended to mistakenly select medium-frequency (LPF4) stimuli as matching high-frequency (Orig) stimuli. These differences are consistent with the idea that cultural groups differ in their dependence upon and attention to different spatial frequency bands (Blais et al., 2021; Tardif et al., 2017), with Americans utilizing higher spatial frequency information relatively more than East Asians and vice versa for lower spatial frequency information.

**Table 7:** Paired comparisons for the confusion matrix data (for Orig items)

Stimulus	Response	Estimate	*SE*	*t* ratio	*p* value
Orig	Orig	− 0.081	0.040	− 2.034	0.047
Orig	LPF4	0.075	0.038	1.986	0.052
Orig	LPF8	0.006	0.016	0.348	0.729

*df* = 58. *p* value adjustment: Bonferroni corrected for nine comparisons. A positive estimate means that East Asians had a larger value than Americans; a negative estimate means the opposite

Our analysis of the confusion matrices shown in Table 6 began with an omnibus ANOVA, in which group (Americans, East Asians) and condition (Orig, LPF4, LPF8) were factors. There was a significant main effect of condition, *F*(8, 464) = 256.80, *p* <. 001, η_g_^2^ = 0.82, but no Group × Condition interaction, *F*(8, 464) = 1.20, *p* = 0.297, η_g_^2^ = 0.02. A Greenhouse–Geiser correction was applied to *p* values. (A main effect of group is not meaningful to compare because responses within each condition sum to 1.)

Our preregistered hypotheses implicated only a subset of the confusions in Table 6. Specifically, we predicted that Easterners would choose more low spatial frequency images whereas Americans would choose more high spatial frequency images. Therefore, we followed the omnibus ANOVA with paired comparisons, Bonferroni corrected for nine comparisons. The results for the unfiltered, Orig stimuli as targets are shown in Table 7. American subjects are significantly more likely than East Asian subjects to correctly recognize Orig target stimuli as what they were—namely, Orig stimuli. This difference between the two group’s conditional probabilities was 8% (*p* = 0.047). In contrast, East Asians were 7.5% more likely to misidentify unfiltered Orig target stimuli as LPF4 stimuli (*p* = 0.052). Only those two contrasts approach or exceed statistical significance (see Supplemental Materials for all comparisons). It is important to appreciate that no other group contrast was even as large as 3%.

## Discussion

Before examining differences between our North American and East Asian subjects, it is essential to consider how the spatial structure of images influences short-term visual memory. Our findings demonstrate that subjects were highly accurate in recognizing target images that retained most of their high-spatial frequency content, as seen in the accuracy for Orig and LPF4 images (Fig. 4). These results align with previous studies showing highly accurate memory for complex visual stimuli (Brady et al., 2008; Standing, 1973). Remarkably, in our study, high-fidelity memory representations were generated from target images viewed for just 2 s before being masked.

In terms of prior research on memory qualities, our results converge with those of Rivera-Aparicio et al. (2021) in terms of showing an advantage in short-term memory for high-resolution images. Extending prior research by using grayscale images and a match-to-sample paradigm, we found that the drop-off in accuracy was largest between moderate amounts of low-pass filtering (LPF4) and high amounts of low-pass filtering (LPF8). Comparisons of confusions, however, indicate that images with a moderate amount of low-pass filtering (LPF4) tended to be confused with images with high amounts of low-pass filtering (LPF8) rather than with those that had not been filtered (Orig). These results suggest prioritization of high-quality information in short-term memory, in terms of both high levels of accuracy as well as potentially treating these types of images as categorically distinct from those with substantial blur; after some amount of blur is present, people have difficulty distinguishing the amount of blur. These results provide further evidence that the pattern of fading vividness identified in Cooper et al. (2019) might be limited to long-term memory and may not characterize short-term memory.

In contrast, performance declined significantly for target images that underwent the most low-pass filtering (LPF8), with a mean proportion correct of only 0.46, barely above the chance level of 0.33. Despite the seemingly subtle differences in high-frequency content between LPF4 and LPF8 images, as shown in Fig. 1, these differences were reflected in SSIM values and were sufficient to produce distinct patterns of confusion. These findings indicate that short-term visual memory for scenes is sensitive to spatial filtering, with modest variations in test images leading to reliable effects on memory.

### Cultural differences in reliance on spatial frequency information

Our study also explored whether cultural differences impact short-term memory for spatial frequency content, drawing on the idea that Westerners prioritize higher spatial frequency information more than Easterners (Blais et al., 2021). We hypothesized that Americans would outperform East Asians in remembering images with high spatial frequency content. The results shown in Table 6 confirmed that cultural differences occur: Americans were significantly more accurate than East Asians in recognizing unfiltered Orig scenes. Planned comparisons (Table 7) indicate a trade-off between higher levels of accuracy for Orig items (for Americans) and more confusions with LPF4 images (for East Asians). However, all group differences disappeared for LPF4 and LPF8 images, suggesting that top-down factors, including cultural influences, contribute to how spatial frequency information is utilized (Flevaris et al., 2011; Shulman & Wilson, 1987; Sowden & Schyns, 2006).

Although our findings extend previous research on cultural differences in prioritizing spatial frequency content (Blais et al., 2021; Caldara et al., 2010; Estéphan et al., 2018; Im et al., 2017; Kelly et al., 2010; Miellet et al., 2013; Rodger et al., 2010; Tardif et al., 2017) to short-term memory tasks involving scenes, these cultural differences were modest compared to the effects of low-pass spatial filtering, regardless of subjects’ cultural background. One possible cause of this effect is that detailed, high-frequency information is critical for identifying specific objects and understanding a scene’s finer semantic details. Although this loss would have a negligible effect on the ability to extract a scene’s gist (Oliva & Torralba, 2001), it becomes important in a challenging situation like ours, when subjects are confronted with a test array in which all alternatives share the same gist. Under those conditions, the availability of high-frequency information is critical (Lowe, 2004).

### Limitations and considerations

Several factors may have limited our study’s relatively modest cultural differences. Our East Asian subjects were international students studying in the USA, and living abroad as they had, even for an average of 2 years, may have sufficed to shift their cultural orientation toward a more North American perspective. How did the time the East Asian subjects spent in the West and their experiences there influence their perceptual and attentional strategies? We tried to restrict East Asian subjects’ degree of acculturation to the USA by restricting our sample to people who had lived no more than 5 years outside their home country. However, for young adults like our subjects, that time is a large fraction of their lives.

Past research suggests that exposure to a novel cultural environment can influence perceptual strategies. For instance, Ueda and Komiya (2012) found that priming participants with culturally specific visual scenes influenced their eye-movement patterns. These results indicate that even brief, short-term exposure to different cultural environments can affect visual attention strategies. While long-term cultural immersion may lead to broader shifts in perceptual processing, further empirical studies are needed to substantiate this claim. Importantly, perceptual strategies can shift over relatively short periods; exposure to different environments or tasks—even for minutes to weeks—can influence attentional strategies and perceptual processing. Because our East Asian participants had spent an average of more than 2 years in the USA, it is possible that their visual processing had already significantly adapted to a Western environment, contributing to the small cultural differences observed in our study.

So, future research could profitably examine whether a shorter residence in the West would boost the difference in reliance on high-frequency details and whether a far longer residence would eliminate the difference. Of course, the amount of time spent outside one’s home country is just one factor contributing to an individual’s degree of acculturation. That time-based measure is mute about the intensity of exposure to and engagement with Western influences—such as films, websites, and everyday visual materials—all of which could affect the cultural biases in local versus global attention reported in previous studies. Examining individual differences in how acclimating to a new culture affects cognition is a promising direction for future research, extending research on the use of long-term memory strategies (Gilliam & Gutchess, 2025) to investigate visual and short-term memory processes.

Additionally, individuals who self-select to study abroad might already share values and cultural orientations similar to Americans (Kitayama et al., 2006). Notably, there were no significant differences between our groups in basic visual measures, such as visual acuity and contrast sensitivity, reducing the likelihood that initial visual processing differences contributed to the observed results. However, the exact factors promoting the group differences remain unclear. Possible explanations include differences in visual environments or cultural factors like congenital myopia, which could influence visual attention habits (Gutchess & Sekuler, 2019).

### Effects of memorability on performance

Using a metric from Isola et al. (2011), we also examined the impact of image memorability on performance. Our selection of test materials excluded very high memorability images, notably ones containing people or cars. However, Table 3 shows that memorability did not significantly affect performance. Several factors may have contributed to this null effect. First, the short time interval between target and test images in our protocol (a few seconds) may have minimized the impact of memorability, as defined by Isola et al. (2011), with a different protocol and much longer retention intervals. Second, apart from difference in high frequency content, all three test mages on each trial in our protocol were similar to the trial’s target image. This considerable within-trial homogeneity may have worked against finding an effect of memorability. Finally, our task might have been performed by extracting local image statistics, bypassing variables represented in memorability measurements. Watson and Ahumada (2011) showed that when an image contains one or two distinct edges, blur discrimination can be driven by a local computation of contrast energy. Although this local approach is not perfectly extensible to natural images like the ones we used, we cannot exclude the possibility that participants may have exploited particular edges or patches in our images rather than judging global variables that would contribute to image memorability. Further research, perhaps with eye tracking or selective masking could help clarify whether performance in our task was based on limited regions of test images or required information from broader, potentially image-wide regions.

### Future directions

In light of the modest cultural effects we observed, future research might investigate how varying durations of cultural immersion influence spatial frequency processing. One approach would be to compare recent East Asian arrivals to the USA (e.g., less than 6 months) with those who have resided in Western environments for multiple years.

Research could also extend our work by exploring the effects of spatial filtering and cultural influences over longer memory delays, as memory’s spatial frequency content is known to change over time (Harvey, 1986). Additionally, testing the impact of filtering lower or medium spatial frequencies could provide further insights into the role of spatial frequency information in memory and how this may vary across cultures. Continuing to investigate the basis for cultural effects is crucial, especially given our findings that individual differences in self-construal or reasoning styles did not account for the observed cultural differences. Exploring the influence of perceptual affordances and scene content, such as the number of objects in different environments (Miyamoto et al., 2006), could shed light on how a culture’s physical environment shapes perceptual biases and memory.

## Conclusion

In conclusion, this research demonstrates that short-term visual memory preserves detailed spatial frequency content, enabling discrimination of even very small variations between stimuli. Memory performance was significantly poorer for images with more low-pass filtering; images filtered the most were rarely confused with unfiltered originals. We found that cultural background modestly affected the use of spatial frequency information, possibly because of perceptual acculturation and task-related factors—namely, the low-level perceptual discrimination demands inherent in our DMS task. Perceptual discrimination can be described as a low-level perceptual process, more complex than detection but still simpler than higher-level processes like recognition, identification, or categorization. These findings underscore the importance of spatial structure in short-term visual memory and highlight the role of cultural influences, albeit modestly, in shaping memory for visual scenes.

## Supplementary Information

Below is the link to the electronic supplementary material.

Supplementary file1 (DOCX 14 KB)

## Data Availability

Data are available at OSF (https://osf.io/hyk6w/). The original versions of picture stimuli are available through the SUN database (https://groups.csail.mit.edu/vision/SUN/hierarchy.html).

## Code availability

R markdown files are available online (https://osf.io/hyk6w/).

## Declarations

### Open Practices Statement

Data and analysis files are available online (https://osf.io/hyk6w/). The study was preregistered (https://osf.io/2w3ht), although the final analytic plan expanded upon that in the initial preregistration.
