# Supplementary material for: Visual short-term memory, culture, and image structure
Source: Atten Percept Psychophys. 2025 May 27;87(5):1630–41. doi: 10.3758/s13414-025-03094-7 (PMC12204929; doi:10.3758/s13414-025-03094-7)
Supplement: Supplementary file 1 — Supplementary file1 (DOCX 14 KB) [file 13414_2025_3094_MOESM1_ESM.docx]

Supplemental materials

Supplemental Table 1. Paired comparisons for the confusion matrix data (expansion of Table 7).

| **Stimulus** | **Response** | **Estimate** | **SE** | **t-ratio** | **p-value** |
| --- | --- | --- | --- | --- | --- |
| Orig | Orig | -0.081 | 0.040 | -2.034 | 0.047 |
| Orig | LPF4 | 0.075 | 0.038 | 1.986 | 0.052 |
| Orig | LPF8 | 0.006 | 0.016 | 0.348 | 0.729 |
| LPF4 | Orig | -0.026 | 0.023 | -1.128 | 0.264 |
| LPF4 | LPF4 | 0.015 | 0.041 | 0.373 | 0.711 |
| LPF4 | LPF8 | 0.010 | 0.039 | 0.267 | 0.791 |
| LPF8 | Orig | 0.008 | 0.012 | 0.668 | 0.507 |
| LPF8 | LPF4 | 0.008 | 0.046 | 0.167 | 0.868 |
| LPF8 | LPF8 | -0.015 | 0.045 | -0.341 | 0.734 |

*df* =58. *p* value adjustment: Bonferroni corrected for nine comparisons. A positive estimate means that East Asians had a larger value than Americans; a negative estimate means the opposite.
